# Evaluation of the Antioxidant Properties and Biological Effects of a Novel Combined Barberry Root–Propolis Extract on HEK293T Cells

**DOI:** 10.3390/ph18010027

**Published:** 2024-12-28

**Authors:** Dana Marcinčáková, Nikola Hudáková, Michal Miłek, Mária Kolesárová, Małgorzata Dżugan, Dasa Cizkova, Jaroslav Legáth

**Affiliations:** 1 Department of Pharmacology and Toxicology, University of Veterinary Medicine and Pharmacy in Kosice, Komenského 73, 041 81 Kosice, Slovakia; dana.marcincakova@uvlf.sk (D.M.); maria.kolesarova@uvlf.sk (M.K.); jaroslav.legath@uvlf.sk (J.L.); 2 Centre of Experimental and Clinical Regenerative Medicine, Small Animal Clinic, University of Veterinary Medicine and Pharmacy in Kosice, 041 81 Kosice, Slovakia; dasa.cizkova@uvlf.sk; 3 Department of Chemistry and Food Toxicology, Institute of Food Technology and Nutrition, University of Rzeszow, Ćwiklińskiej 1a St., 35-601 Rzeszow, Poland; mmilek@ur.edu.pl (M.M.); mdzugan@ur.edu.pl (M.D.); 4 Institute of Neuroimmunology, SAS, 845 10 Bratislava, Slovakia

**Keywords:** propolis, barberry, berberine, antioxidant, HPTLC, cell proliferation, metabolic activity, migration

## Abstract

**Background/Objectives:** The health benefits of honeybee products and herbs are well known, and their appropriate combination may enhance their biological efficacy. This study investigated the biological properties of a combined barberry root and propolis extract (PBE) in comparison to a propolis extract (PE), a barberry root extract (BE), and pure berberine (BN). **Methods:** The antioxidant properties were evaluated using DPPH and FRAP methods and total phenolic contents (TPC) were assessed by the Folin–Ciocalteu method. HPTLC was used to quantify the BE in the tested samples. Their effect on HEK293T cells was monitored in real-time by using the xCELLigence system which recorded changes in the proliferative activity (PA). The metabolic activity (MA) was evaluated using an MTS test and cell migration was analyzed via a scratch assay. **Results:** The PE exhibited a higher TPC (198.67 mg/g) than the BE (119.3 mg/g). The PBE exhibited a comparable antioxidant effect to that of the PE. In the cell assays, the PE, the BE, and BN significantly reduced the proliferative activity at higher concentrations (*p* < 0.0001) while the PBE demonstrated a lower cytotoxicity and proved to be safer for the tested cells. The highest IC_50_ value was determined for the PBE (130 µg/mL), suggesting that this combination has a reduced cytotoxicity. However, the scratch test did not confirm a significant supportive effect of the PBE on cell migration. **Conclusions:** Although the PBE did not show enhanced antioxidant properties, it may mitigate cytotoxicity and support proliferation at lower concentrations. This suggests that extraction of raw propolis with a previously prepared barberry extract results in a safer preparation, but its therapeutic potential requires further studies using biological models.

## 1. Introduction

In the era of many human diseases and the increasingly common phenomenon of drug resistance, new medicinal substances are constantly sought. In recent years, there has been a return to substances of natural origin isolated from natural sources of active substances or complex extracts [1]. Medicinal plants are the most commonly used; however, bee products such as honey, propolis, royal jelly, bee venom, bee pollen, and bee bread have also demonstrated great pharmaceutical potential [2,3]. Due to the unique properties of honey, the use of a combination of bee products with herbs is not a new idea; historical sources prove that the effectiveness of such combinations has been understood by humans since ancient times [4,5,6]. However, most of these recipe types and complex medicines involve combinations of herbs with honey; the idea of creating new formulations based on propolis and herbs is relatively new.

The general composition of propolis is as follows: the majority are resinous and balsamic substances (up to 70%), the wax content ranges from a few to a dozen or so percent (less frequently over 20%), and volatile substances usually constitute up to 1% (in the case of specific types of propolis there may be even up to 8.5%) [7]. The detailed phytochemical profile depends on the botanical sources used by bees and includes numerous compounds from the groups of polyphenols (phenolic acids, flavonoids, phenylpropanoids, stilbenes), terpenoids, esters, fatty acids, and hydrocarbons [8,9]. The chemical profile of propolis extracts depends on the extraction solvent type, solvent ratio, and extraction procedure [10]. The main biological effects of propolis and its extracts include antioxidant, antimicrobial, antiviral, anti-inflammatory, and anticancer effects [8,9]. Possible mechanisms of the anticancer effect of propolis and its constituents include induction of apoptosis, induction of autophagy, anti-proliferative action and cell cycle arrest, inhibition of angiogenesis and metastasis, suppression of the immune response, and epigenetic mechanisms [11,12].

Among the numerous medicinal plants used in the pharmaceutical industry, the genus *Berberis* has recently become very popular. This genus includes several hundreds of species of shrubs distributed mainly in Asia, Europe, North Africa, and North America [13,14]. Various species of barberry are cultivated worldwide for decorative purposes but also as a source of pharmaceutical raw materials which include almost all parts of the plant such as the roots, root bark, twigs, leaves, and fruit [13]. The main barberry species of pharmacognostic importance is *B. vulgaris* L. which is a typical alkaloid raw material. It contains a full set of isoquinoline alkaloids including berberine, oxyberberine, lambertine, magniflorine, berbamine, palmatine, columbamine, and jatrorrhizine [13,14]. Additionally, secondary metabolites of barberry include flavonoids, anthocyanins, and carotenoids [13]. The dominant alkaloid of barberry is berberine which is also present in other plants mainly from the *Ranunculaceae*, *Rutaceae*, and *Berberidaceae* families [15]. Literature reviews have described a wide spectrum of biological effects of berberine: antioxidant, anti-inflammatory, anticancer, antidiabetic, and antimicrobial [15,16]. Studies on cancer cells have shown the possibility of including berberine as a part of cancer therapy due to the inhibition of telomerase, induction of apoptosis, involvement in reactive-oxygen-species-mediated mechanisms, and antimetastatic, antiproliferative, and anti-invasive effects [16].

The main aim of this work was to evaluate the biological properties of a unique combined extract; propolis extracted using a previously prepared aqueous-ethanolic extract of barberry root. The antioxidant properties, polyphenol contents, and in vitro effect of a novel extract (propolis–barberry root extract; PBE) on a cell model were compared with propolis extract (PE), barberry root extract (BE), and pure berberine (BN). The research hypothesis assumes that propolis extraction using a plant extract will allow for the obtainment of preparation with enhanced positive effects due to the interactions of the propolis and barberry components.

## 2. Results and Discussion

### 2.1. TPC and Antioxidant Capacity

TPC and the antioxidant capacity were assessed for freeze-dried extracts. The results are shown in Table 1.

The prepared PE contained 198.67 mg/g of phenolic contents expressed as gallic acid equivalents which is a typical value for extracts of Polish poplar propolis prepared in this manner. Similarly, slightly higher results (252.38–326.34 mg/g) were obtained earlier for nine propolis samples from the same region of Poland [17]. The composition and activity of propolis are strongly influenced by its quality as well as environmental factors such as climate, flora composition, and harvest time [9]. The quantity of phenolic contents in ethanol extracts of Polish propolis determined earlier by Moskwa et al. was also comparable (137.19 to 275.79 mg/g) [18,19]. In addition, the antioxidant properties tested via two different methods using two different mechanisms are similar to the levels obtained for the previously tested ethanolic propolis extracts [17]. In the BE, 119.3 ± 21.24 mg GAE/g of phenolic content per gram was determined. This value is higher than that provided in the literature, e.g., for the roots of *B. vulgaris* collected in Croatia where a content of 7.29–10.34 mg/g was reported [20]. In this case, discrepancies may result from the use of different extraction conditions or the quality of the raw material itself. Surprisingly, after the extraction of propolis, the use of BE did not result in the enhancement of the antioxidant effect nor the polyphenol content in the obtained PBE preparation. The obtained results did not differ significantly (*p* > 0.05) from the values for the PE extract obtained using 70% ethanol instead of BE. The lack of at least an additive effect can be explained by interactions between the components of BE and raw propolis. Due to the resinous structure of propolis, it can be assumed that some of the BE components were adsorbed by propolis which in turn reduced the extraction efficiency of the bioactive components of propolis.

### 2.2. HPTLC Berberine Determination

**Figure 1:**
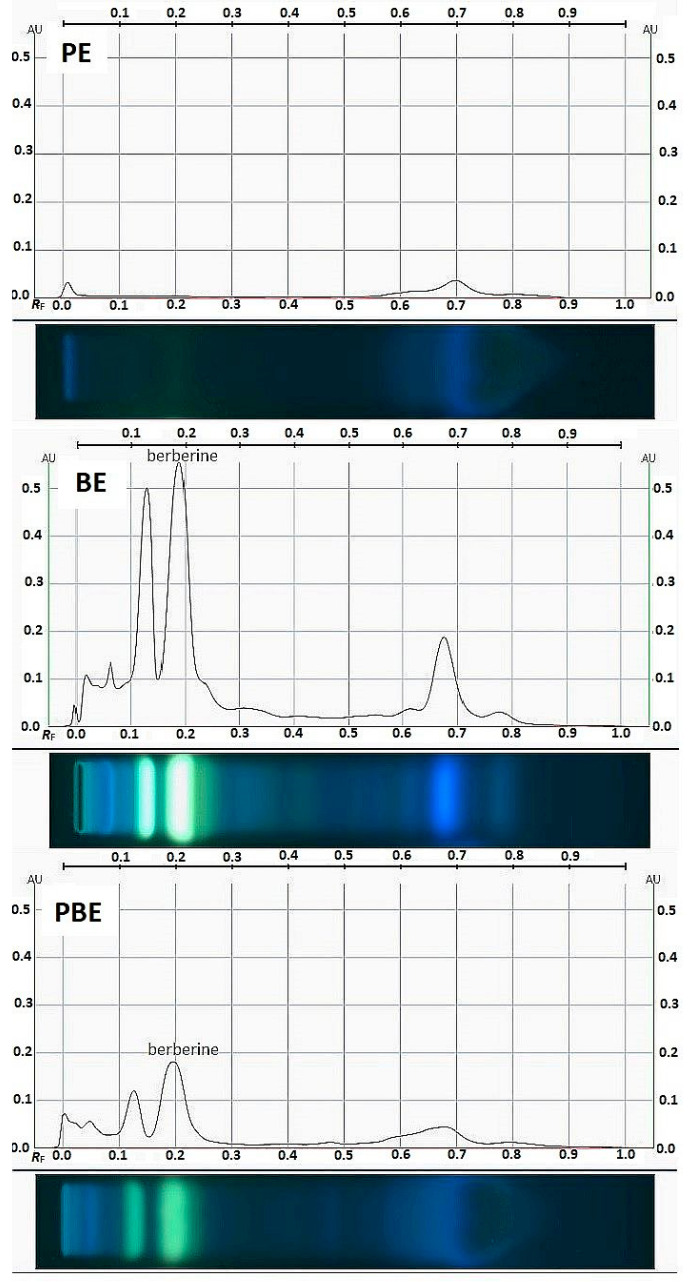
HPTLC profiles of the tested extracts (50 mg/mL) visualized in UV 366 nm. PE—propolis extract, BE—barberry root extract, PBE—propolis–barberry root extract.

Barberry root (like all other parts of the plant) is a typical alkaloid raw material [14,21]. The tested extracts were analyzed by High-Performance Thin Layer Chromatography (HPTLC) to estimate the content of the main alkaloid BN. An assessment of the berberine content is usually performed by HPLC; however, HPTLC has been used more recently [22,23,24]. This technique is less cost- and time-consuming; moreover, it allows for the direct comparison of samples during a single analysis. Figure 1 shows images of the HPTLC tracks and chromatograms for the tested extracts.

Chromatographic separation conducted on an HPTLC plate revealed two main bands which fluoresced yellow under a 366 nm UV light at Rf 0.14 and 0.19. The second of these bands, based on a comparison with the standard, was assigned to BN. Moreover, blue bands were also visible which may have come from other unidentified compounds; it is particularly visible in the case of the PE for which the blue bands remained unresolved in the high Rf range. The pattern of two main bands originating from alkaloids is typical for extracts from barberry root [25,26,27]. Thanks to calibrating for berberine sulfate, the content of this alkaloid in the extracts was determined. The BE contained 111.06 mg/g of BN (11.1%) which is consistent with the literature that reports the content of this alkaloid in *B. vulgaris* root extracts to be at a level of 7–12% [20,28]. The BN content in the PBE was much lower and amounted to 1.48 mg/g. These results confirm our previous speculations about the absorption of BE components in the resinous propolis matrix.

### 2.3. The In Vitro Functional Effect on HEK293T Cells

#### 2.3.1. Monitoring the Cell Response via the Real-Time–xCELLigence System

The biological effect of the tested substances was evaluated using an analyzer of cell behavior in real-time, the xCELLigence system (real-time cell analyzer; RTCA, Acea Bioscience, CA, USA). This system allows the continuous observation of cell behavior, adherence, and proliferative activity (PA) through impedance measurements that are expressed as cell index values (CI) and recorded into curves. This enables the tracking of cellular responses dynamically over time, providing insights into substance effects at multiple time points rather than relying solely on endpoint analyses [22]. RTCA has been previously used by many authors for drug screening [23], toxicology studies [24,29], and virology research [30].

**Figure 2:**
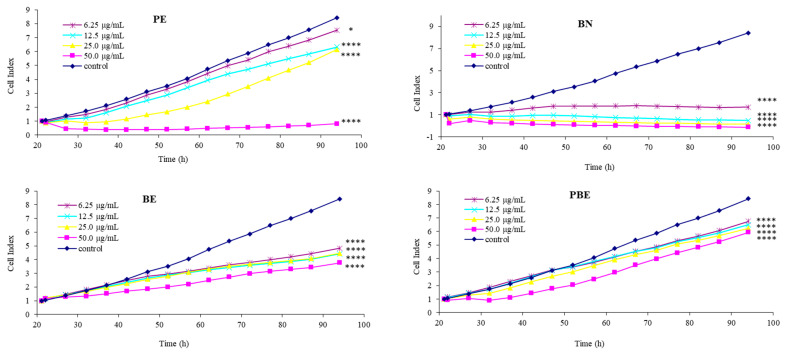
Changes in cell index after treatment with PE—propolis extract, BE—barberry root extract, BN—berberine, PBE—propolis–barberry root extract at tested concentrations (6.25, 12.5, 25.0, and 50.0 µg/mL) in comparison to a control group without treatment. Statistical processing of the results at the end of treatment (72 h) is shown as significance compared to the control group (one-way ANOVA followed by post hoc Dunnett’s test: * *p* < 0.05, **** *p* < 0.0001).

The functional effect of the tested samples of PE, BE, PBE, and BN on normal epithelial HEK293T cells was evaluated during a 72 h treatment. The PA of the treated cells was recorded in real-time via the xCELLigence system (shown in Figure 2). At all tested concentrations, the PE, the BE, and BN caused a significant dose-dependent drop in the PA during the entire experiment (*p* < 0.05; *p* < 0.0001). On the other hand, the PA was not reduced by the PBE combination at lower concentrations (6.25 and 12.5 µg/mL) after 24 h of exposition (*p* > 0.05). The decline in the PA was weaker but significant (*p* < 0.001) in comparison to the PA of cells treated with other tested substances after 48 and 72 h of treatment with the PBE combination.

In our experiment, no significant increase in cell proliferation was observed for any of the tested samples and concentrations compared to the control. The effect of propolis on the PA was marginally demonstrated in cells treated with PE at lower concentrations (6.25 and 12.5 µg/mL) where the trend of the PA was similar to that of the control until 48 h of the experiment (*p* > 0.05). The higher concentrations (25.0 and 50.0 µg/mL) caused a drop in the CI (*p* < 0.0001). This finding is consistent with Garcia et al. [31], who found that lower concentrations of propolis can promote the viability and proliferation of human adipose-derived stem cells (ADSC) but that higher concentrations can lead to cytotoxic effects. The cytotoxicity is supposedly attributed to the pro-apoptotic effects and oxidative stress induced by natural extracts when used in excess [32].

Similarly, barberry exerts strong antioxidant properties which, except for positive effects, can also induce cytotoxic effects in various cells. An in vitro study conducted by Ivan et al. [33] showed that barberry extract as well as berberine exerted cytotoxic effects and inhibited the growth of cancer cells (MCF-7, HepG2, and Caco-2) dependent on time and concentration. In addition to *Berberis vulgaris*, extracts from ornamental varieties of *Berberis thunbergii* have recently been tested for their effect on cell viability (HaCaT, A375, and Caco-2) in vitro, demonstrating differences between varieties as well as a dose-dependent effect. However, no clear link between the studied effects of the extracts and their berberine content has been established [34].

Except for monitoring the effects of the tested substances in real-time, the xCELLigence software (vs. 1.2.1) can also calculate the IC_50_ (half-maximal inhibitory concentration) which is a crucial metric in pharmacology indicating the amount of a substance needed to inhibit a biological process by 50%. The *Berberis vulgaris* root extract has been studied for its chemopreventive properties and the IC_50_ value against LS180 cells was 4.31 µg/mL and 46.06 µg/mL against HT-29 cells [35]. It indicates varying degrees of effectiveness dependent on the cell line [36,37]. Similarly, propolis extracts exert different IC_50_ values depending on the specific cell line tested. Teerasripreecha et al. [38] found that the IC_50_ values ranged between 41.3 to 52.4 μg/mL for normal human fibroblast cells (Hs27). These findings are consistent with our results: the IC_50_ for the PE was 63 µg/mL, for the BE 45 µg/mL, and for BN 6.5 µg/mL (48 h). However, we found that the IC_50_ for the combination PBE was even higher (130 µg/mL). These results indicate the mitigation of propolis’s and barberry’s cytotoxicity when combined (PBE).

#### 2.3.2. Evaluation of the Metabolic Activity via an MTS Test

**Figure 3:**
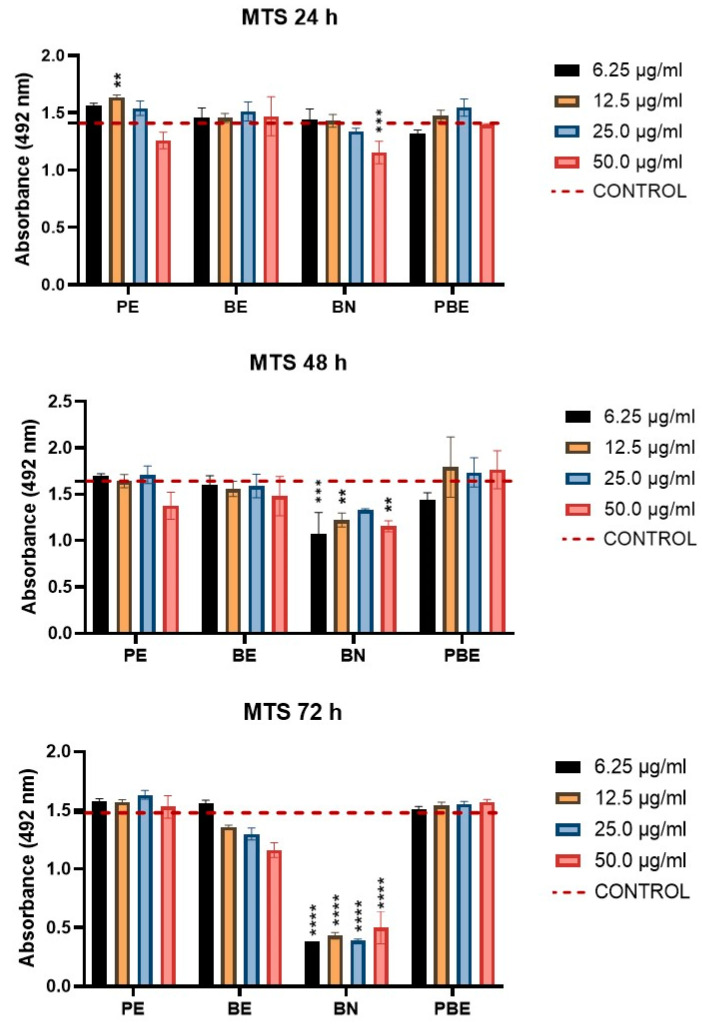
Results of the MTS assay on HEK293T cells after 24, 48, and 72 h. Cells were cultured in media supplemented with PE—propolis extract, BE—barberry root extract, BN—berberine, PBE—propolis–barberry root extract at various concentrations (6.25, 12.5, 25.0, and 50.0 µg/mL) and a standard culture medium in the case of the control. Statistical processing of the results is shown as a significance compared to the control group (one-way ANOVA followed by post hoc Dunnett’s test: ** *p* < 0.01, *** *p* < 0.001, **** *p* < 0.0001). The data is presented as the mean ± SD (n = 3).

The effect of the PE, BE, BN, and PBE on the metabolic activity (MA) of HEK293T cells was evaluated via a standard colorimetric MTS test after 24, 48, and 72 h of application of the extracts (Figure 3).

The results show that BN had the strongest effect on the MA of the cells. Compared to the control, we recorded a significant decrease in metabolic activity (MA) at concentrations of 50 µg/mL (*p* < 0.001) at 24 h, 6.25 (*p* < 0.001), 12.5 (*p* < 0.01), and 50.0 µg/mL (*p* < 0.01) after 48 h and at all observed concentrations of BN (*p* < 0.0001) after 72 h. Regarding BN, experimental studies focusing on the toxic effect of BN reported that exposure of 10 and 30 µM BN to PC12 cells increased the cytotoxicity which was manifested as an increase in apoptotic cell death. In vitro and in vivo studies (10 and 30 µM up to 48 h) with BN against 6-hydroxydopamine (6-OHDA) induced neurotoxicity in rats and PC-12 cells [39]. Moreover, BN has been reported to cause cytotoxicity and adversely influence the synthesis of DNA [39]. Several authors also focused on the in vitro cytotoxicity of propolis; Lopez et al. placed great emphasis on the limited data regarding the cytotoxicity of propolis preparations against non-cancerous human cells. He stated that safe use of red propolis requires reduction in the dose to below 50 µg/mL [37,40]. In our study, the tested PE significantly improved (*p* < 0.01) the MA of the HEK293T cells compared to the control at a concentration of 12.5 µg/mL after 24 h.

Interestingly, the PBE combination at 12.5 (*p* < 0.05) and 25.0 µg/mL (*p* < 0.01) significantly increased the MA compared to the BE after 72 h of exposure. However, we were more interested in studying whether the combined extract (PBE) affects the MA of cells or not. The beneficial effect of propolis combined with several substances has been described in more detail. For example, the combined application of propolis and honey to aid in the healing process of an open wound in rats exhibited a synergistic effect [37]. The combination of propolis nanoparticles (NPro) and photodynamic therapy based on nanocurcumin (NCur-PDT) in the remineralization of WSL ex vivo was also described [41]. Concerning barberry root, a recent study demonstrated that the combination of galangin, one of the flavonoids found in propolis, with berberine synergistically led to the inhibition of cell growth, apoptosis, and cell cycle arrest in the G2/M phase with increased levels of intracellular reactive oxygen species (ROS) in esophageal carcinoma cells [42].

The results quoted, however, refer to the combination of propolis extract with plant extracts immediately before application. In our study, a different approach was used. We combined the bioactive components during propolis extraction using barberry extract (BE). As shown by HPTLC, the bioactive fraction of the PBE is different from the expected additive effect of the PE + the BE. Already in the evaluation of the antioxidants, it is clearly visible that there is no additive effect as the activity of the PBE is comparable to that of the PE and higher than that of the BE. In the evaluation of the biological activity, the effect is BE > PBE > PE which confirms that the PBE extract contains fewer barberry components than BE and the barberry components enhance the effect of the PE. We have, therefore, obtained a preparation better than the PE and the BE which is especially visible in the scratch test.

#### 2.3.3. Comparison of the RTCA and MTS Results

For better visualization and evaluation of the results obtained by the xCELLigence system (CI values) and MTS test (absorbance in nm), all the data obtained were expressed as percentages compared to control cells without treatment (100%). Values are summarized in Table 2.

The results obtained by the xCELLigence system and MTS test were in correlation as indicated by the Pearson correlation coefficient. The value after 24 h was r = 0.507 (moderate correlation), after 48 h r = 0.745 (moderate correlation), and 0.850 (strong correlation) was observed after 72 h of treatment. Overall, in all mentioned time intervals, the value of the Pearson test was 0.518 indicating a moderate positive linear correlation.

#### 2.3.4. Cell Migration

Since cell migration is an important factor involved in many biological processes such as embryological development, new tissue formation, immune response or inflammation, and oncological diseases [43], the study of the migratory response of cells to substances of various origins is important. Using the scratch test, we determined whether the tested extracts could alter the migration of HEK293T cells.

**Figure 4:**
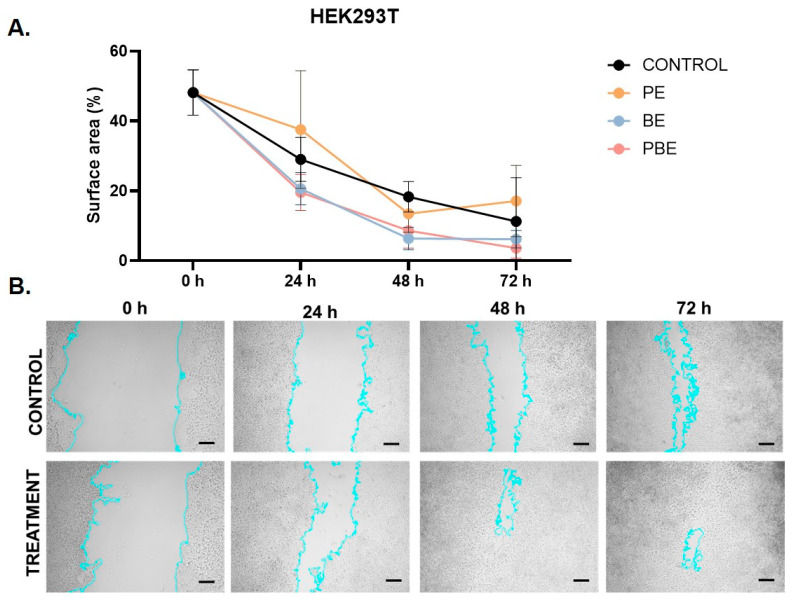
The effects of barberry root and propolis extracts on HEK293T cell migration. Results of the scratch assay on HEK293T cells during which mechanical damage was followed by culturing cells in PE, BE, PBE, and DMEM (control). Photos were taken after 24, 48, and 72 h. (**A**) The graph shows the time-dependent profile of the % of the area not occupied by cells ± SD. (**B**) Representative photos of the scratch test after 0, 24, 48, and 72 h in the control group and in the groups with the largest difference in the area not covered by cells compared to the control group which was the PBE after 24 h, BE after 48 h, and PBE after 72 h. Scale bar: 100 µm.

The results shown in Figure 4 represent the changes in the percentage of the empty area (that is, the area not occupied by cells) at 0, 24, 48, and 72 h after mechanical damage was conducted. In our measurements, we did not notice a significant difference between the control and the experimental groups (PE, BE, PBE) in which the cells were cultured in media with the addition of PE, BE, and PBE. In the BN group, we noticed a significant decrease in cell adhesion after 24 h which made it impossible to quantitatively and statistically evaluate the data from this experimental group. On the other hand, as shown in Figure 4 in the case of the BE and the PBE, we observed an improvement in cell migration into the area not covered by cells after 24, 48, and 72 h compared to the control group despite it not being statistically significant. This difference, however, was not observed in cells treated with the PE. The samples with the best capacity to influence cell migration were the PBE after 24 and 72 h and the BE after 48 h (Figure 4). Many authors have investigated the role of bee products in wound healing at a cellular level. When investigating the effect of propolis on keratinocyte migration, Martinotti et al. proposed H_2_O_2_ as the main mediator of the regenerative properties of propolis [44]. On the other hand, when analyzing the promotion of cell migration of human fibroblasts, Afonso et al. stated that the effect of the combination of propolis and honey is not synergistic and there is only an additive effect of propolis and honey [45]. In our experiment, the positive effect of propolis on cell migration was not confirmed. Interestingly, we recorded the combined PBE as the sample with the best cell migration despite it not being significant (*p* > 0.05).

Several authors describe propolis as a natural substance with nephroprotective properties mediated by several mechanisms. Propolis improves kidney function and reduces the retention of uremic toxins in plasma. It inhibits α-SMA expression and collagen deposition, and disrupts fibrotic epithelial–mesenchymal transition and TGF-β signaling pathways, thereby alleviating fibrosis [46]. Propolis can also improve kidney function thanks to its antioxidant, anti-inflammatory, and anti-apoptotic properties as it modulates oxidative stress, inflammation, and DNA damage [47]. Specifically, studies have demonstrated that propolis possesses strong antioxidant properties which reduce oxidative damage of renal tissues by decreasing lipid peroxidation and oxidative stress markers such as malondialdehyde and reactive oxygens species (ROS) [48,49,50].

As for berberine, the positive effect on kidneys is due to its antifibrotic, anti-inflammatory, and antioxidant properties [51]. It can activate the PI3K/Akt signaling pathway, reduce oxidative stress by increasing antioxidant enzymes (GSH and SOD), and inhibit apoptosis by modulating the expression of Nrf2 and HO-1 [52].

Wound healing is another critical area where propolis exerts beneficial effects. Its anti-inflammatory and antimicrobial properties contribute significantly to the wound-healing process. Al-Waili states that propolis enhances the immune response and reduces inflammation, which is vital for effective wound healing [53].

In conclusion, the innovative combined extract of propolis and barberry root (PBE) demonstrates a higher safety for renal cells. The antioxidant and anti-inflammatory properties of this preparation along with the potential to promote cellular migration suggest that this combination could be beneficial in the management of renal injuries and diseases. However, due to the great variability of raw materials (propolis and barberry root), the results should be considered preliminary, and the research should be repeated with emphasis on the analysis of the phytochemical composition of the obtained preparation. Further research should aim to elucidate the specific mechanisms underlying these effects and to explore the potential clinical applications of this combined propolis–barberry extract.

## 3. Materials and Methods

### 3.1. Materials

Propolis for the study was purchased from a local apiary (Sieklówka, Poland) and barberry root (*Berberis vulgaris*) was purchased from Nat Vita (Długołęka, Poland). All reagents used were of at least analytical grade.

### 3.2. Preparation of Extracts

Barberry root extract (BE) was prepared as follows: 20 g of ground barberry root was mixed with 200 mL of 70% aqueous ethanol (Stanlab, Lublin, Poland) and then sonicated in an ultrasonic bath (Sonic-10, Polsonic, Warsaw, Poland) in two cycles (20 min each, 700 W, max. 50 °C). Part of this crude extract was used to prepare propolis–barberry root extract (PBE): 10 g of crushed propolis was poured into 100 mL of the extract, shaken for half an hour (400 rpm, Benchmark OrbiShaker MP, Benchmark Scientific, Sayreville, NJ, USA), and then left to macerate for 5 days with occasional shaking. Afterward, the extract was filtered through a filter paper. Propolis extract (PE) was prepared analogously using 70% aqueous ethanol as the solvent. Fifteen mL of each crude extract was subjected to ethanol evaporation using a centrifugal evaporator (RVC 2–18 CDPlus, Martin Christ, Osterode am Harz, Germany) and then lyophilized to obtain dry extracts (Alpha 1–2 LD plus, Martin Christ, Osterode am Harz, Germany).

### 3.3. Total Phenolic Content and Antioxidant Capacity

To determine the phenolic content and antioxidant properties, extracts were reconstituted in 70% aqueous ethanol at a concentration of 500 μg/mL. TPC, DPPH antiradical potential, and FRAP-reducing power were determined as described by Miłek et al. [54].

### 3.4. HPTLC Berberine Profile

Berberine content in the tested extracts was determined by the HPTLC method according to Alam et al. [55] with a slight modification [34]. HPTLC Silica Gel 60 F254 plates (20 cm × 10 cm) (Merck, Darmstadt, Germany) were used. Quantitative analysis was performed based on software-generated chromatograms (Vision CATS 3.2, Camag, Muttenz, Switzerland) for plate visualization at UV 366 nm. Berberine sulfate (Aliness, Ostrówiec, Poland) methanolic solution (HPLC-grade methanol, Honeywell, Charlotte, NC, USA) (250 μg/mL) was used for calibration (0.5 to 2 μg per band).

### 3.5. Cultivation of HEK293T Cells

An immortalized human embryonic kidney cell line HEK293T (ATCC CRL-11268) was purchased and kindly provided by the Institute of Neuroimmunology of the Slovak Academy of Sciences in cooperation with the Small Animal Clinic, Veterinary Hospital at the University of Veterinary Medicine and Pharmacy in Košice. The cell culture was maintained in cultivation conditions of 37 °C and 5% CO_2_ in DMEM high-glucose medium (Dulbecco’s Modified Eagle’s Medium High glucose, Sigma Aldrich, Darmstadt, Germany) supplemented with 10% fetal bovine serum (FBS, Sigma, Aldrich, Germany) and 2% antibiotics (Penicillin/Streptomycin/Amphotericin; ATB/ATM, Sigma Aldrich, Darmstadt, Germany). The medium was replaced by a fresh one every 2–3 days.

### 3.6. xCELLigence Real-Time Cell Analysis

The xCELLigence system (Roche Applied Science, Mannheim, Germany) is a commercial microfluidic system that enables continuous monitoring of cell adherence in vitro. In this work, xCELLigence 1.2.1 software was used. This system is based on an established electronic impedance (relative resistance). The wells in which the cells are cultured are equipped at the bottom with microelectrodes that connect to the cultured cells through sensors and record resistance changes. Microelectrodes are embedded in special 16-well E-plates (Acea Bioscience, Mannheim, Germany). The measured impedance changes are evaluated as the so-called cell index (CI), which represents differences in the number of cells, adherence, morphology, viability, and proliferation [23]. In the absence of live or proliferating cells, CI values are close to zero. Shortly, the first step was the addition of 100 μL of medium to the wells of three E-plates for a background measurement. After equilibration at 37 °C and 5% CO_2_, the E-plates were inserted into the xCELLigence impedance measurement system. After ensuring that all junctions function within the specified measurement range, cells were seeded (5 × 10^3^ cells per well). After the initial 22 h of cell cultivation, solutions of PE, BE, BN, and PBE at final concentrations of 6.25, 12.5, 25.0, and 50.0 µg/mL were added to the cells. Cells without treatment served as the control. The xCELLigence software recorded CI values every hour (94 h) and displayed them in curves. IC_50_ values for the tested extracts were determined using the xCELLigence software for the 48th hour of treatment.

### 3.7. MTS Test

To determine the effect of the tested substances on the MA, a colorimetric MTS assay using an MTS kit (Invitrogen Inc, Carlsbad, CA, USA) was used. This test was performed simultaneously with RTCA monitoring and the cells from the same passage were used. Cells were seeded into three 96-well plates (Greiner-bio-one, Kremsmünster, Austria) at a density of 8 × 10^3^ cells/well and cultured in a standard culture medium (DMEM High Glucose + 10% FBS + 2% ATB/ATM) at 37 °C and 5% CO_2_. After 24 h, the medium was replaced with 100 μL of media with PE, BE, BN, and PBE at final concentrations of 6.25, 12.5, 25.0, and 50.0 µg/mL. Cells in the standard medium were used as a negative control. After 24, 48, and 72 h, 10 μL of MTS reagent was added. At the end of the incubation period, the absorbance was measured at 450 nm using a Synergy HT spectrophotometer (Biotek, Winooski, VT, USA). The data was statistically evaluated and recorded into graphs.

### 3.8. Scratch Assay

A scratch assay was used to determine the rate of cell migration. Briefly, cells were seeded into a 24-well tissue culture plate at a density of 400,000 cells/well in DMEM culture medium. After the cells reached ~80–90% confluence as a monolayer, the medium was removed from each well. A 200 μL pipette tip was used to scrape the cell monolayer in a straight line to create a scratch. Cell debris was removed by washing the cells with phosphate buffered saline (PBS, Sigma Aldrich, Darmstadt, Germany) and replaced by DMEM (Dulbecco’s Modified Eagle Medium) for the control group and PE, BE, and PBE solutions for the experimental groups. The scratch assay was performed in triplicate wells. Seven pictures per group were taken after 0, 24, 48, and 72 h after scraping the cells. Pictures were taken by an inverted microscope Zeiss Axiovert 200 (Zeiss, Oberkochen, Germany) and the ImageJ FIJI Wound Healing Tool was used to measure the surface area of the wounds.

### 3.9. Statistical Evaluation

Statistical analyses were performed using GraphPad Prism software 10.3.1 (GraphPad, La Jolla, CA, USA) with the statistical significance set at *p* < 0.05. To compare the continuous variables between group means, we used ANOVA with Tukey’s and Dunnett’s post hoc corrections. The results obtained were expressed as the mean ± standard deviation (n = 3). The intensity of the correlation between the results of the RTCA analysis and the MTS test was determined using the Pearson correlation coefficient.

## Figures and Tables

**Table 1:** TPC and antioxidant capacity of the tested extracts.

	TPC [mg GAE/g]	FRAP [μmol TE/g]	DPPH [μmol TE/g]
PE	198.67 ± 11.51 ^b^	618.70 ± 20.88 ^b^	398.22 ± 8.17 ^b^
BE	119.30 ± 21.24 ^a^	450.72 ± 23.25 ^a^	243.80 ± 65.75 ^a^
PBE	194.31 ± 8.12 ^b^	626.81 ± 30.94 ^b^	366.42 ± 24.71 ^b^

PE—propolis extract, BE—barberry root extract, PBE—propolis–barberry root extract. ^a,b^—means that the columns marked with different superscript letters are significantly different (*p* < 0.05). The data is presented as the mean ± SD.

**Table 2:** A table summarizing the changes in the proliferative and metabolic activity of HEK293T cells treated with the tested substances expressed as percentages.

	24 h		48 h		72 h	
	PA (%)	MA (%)	PA (%)	MA (%)	PA (%)	MA (%)
PE (µg/mL)						
6.25	91.20 ± 5.29	110.48 ± 1.66	92.47 ± 2.71	102.53 ± 1.58	89.61 ± 1.37 *	103.56 ± 1.52
12.5	79.97 ± 3.71 ****	115.79 ± 1.49 **	80.89 ± 0.27 ****	99.37 ± 4.30	75.04 ± 0.13 ****	103.15 ± 1.35
25.0	46.08 ± 5.77 ****	109.01 ± 4.50	56.09 ± 2.33 ****	103.42 ± 5.74	72.27 ± 4.37 ****	107.27 ± 2.39
50.0	13.14 ± 4.35 ****	89.13 ± 5.12	9.27 ± 2.78 ****	83.27 ± 8.77	9.61 ± 3.14 ****	100.54 ± 6.35
BE (µg/mL)						
6.25	91.74 ± 4.98	103.44 ± 5.76	66.01 ± 4.86 ****	97.19 ± 5.69	57.34 ± 4.83 ****	102.75 ± 1.45
12.5	86.38 ± 4.82 **	103.06 ± 2.83	62.32 ± 4.56 ****	94.19 ± 5.00	51.94 ± 4.51 ****	89.26 ± 1.06
25.0	83.32 ± 4.21 **	107.05 ± 5.83	64.00 ± 2.84 ****	96.15 ± 7.65	52.29 ± 1.49 ****	85.34 ± 3.32 *
50.0	61.68 ± 1.24 ****	110.57 ± 4.14	50.81 ± 1.48 ****	89.51 ± 12.90	44.35 ± 0.34 ****	76.29 ± 4.17
BN (µg/mL)						
6.25	59.25 ± 6.19 ****	102.18 ± 6.55	32.20 ± 4.92 ****	65.22 ± 9.71 ***	20.27 ± 3.39 ****	24.97 ± 0.00 ****
12.5	33.20 ± 4.95 ****	101.22 ± 3.90	11.87 ± 1.41 ****	73.95 ± 3.27 **	5.70 ± 0.13 ****	28.32 ± 1.31 ****
25.0	15.46 ± 1.15 ****	94.88 ± 1.81	4.92 ± 0.61 ****	80.45 ± 0.57	1.69 ± 0.34 ****	25.69 ± 0.53 ****
50.0	4.69 ± 0.28 ****	81.71 ± 6.91 ***	0.70 ± 0.72 ****	69.81 ± 2.51 **	1.58 ± 0.82 ****	32.92 ± 6.44 ****
PBE (µg/mL)						
6.25	102.74 ± 9.42	93.26 ± 2.35	84.27 ± 9.57 ***	87.12 ± 4.70	79.61 ± 8.38 ****	99.38 ± 1.30
12.5	100.29 ± 2.44	104.36 ± 3.46	83.20 ± 3.97 ***	108.48 ± 19.68	77.02 ± 4.77 ****	101.42 ± 1.85
25.0	87.08 ± 2.41 *	109.51 ± 5.35	79.23 ± 3.29 ****	105.01 ± 9.56	73.63 ± 3.32 ****	102.12 ± 1.48
50.0	56.25 ± 2.02 ****	98.88 ± 0.55	66.27 ± 3.00 ****	106.83 ± 12.46	69.75 ± 4.60 ****	103.17 ± 1.50
control	100.00 ± 4.74	100.00 ± 4.50	100.00 ± 6.79	100.00 ± 1.73	100.00 ± 8.18	100.00 ± 7.73

PE—propolis extract, BE—barberry root extract, BN—berberine, PBE—propolis–barberry root extract. Results are expressed in % in comparison to a control group without treatment (100%). Statistical processing of the results is shown as a significance compared to the control group (one-way ANOVA followed by post hoc Dunnett’s test: * *p* < 0.05, ** *p* < 0.01, *** *p* < 0.001, **** *p* < 0.0001). Data is presented as the mean ± SD (n = 3).

## Data Availability

Data is contained within the article.
